# Trends in Obstetrics and Gynecology Residency Applications in the Year After Abortion Access Changes

**DOI:** 10.1001/jamanetworkopen.2023.55017

**Published:** 2024-02-07

**Authors:** Maya M. Hammoud, Helen K. Morgan, Karen George, Arthur T. Ollendorff, John L. Dalrymple, Dana Dunleavy, Min Zhu, Erika Banks, Bukky Ajagbe Akingbola, AnnaMarie Connolly

**Affiliations:** 1 Department of Obstetrics and Gynecology, University of Michigan Medical School, Ann Arbor; 2 Larner College of Medicine at University of Vermont, Burlington; 3 Virginia Tech Carilion School of Medicine, Roanoke; 4 Harvard Medical School, Boston, Massachusetts; 5 Association of American Medical Colleges, Washington, DC; 6 NYU Long Island School of Medicine, New York, New York; 7 Department of Obstetrics and Gynecology, University of Minnesota, Minneapolis; 8 American College of Obstetricians and Gynecologists, Washington, DC

## Abstract

**Importance:**

State-specific abortion restrictions currently affect the training of approximately 44% of obstetrics and gynecology (OBGYN) residents in the US. Examination of where future trainees apply for residency is important.

**Objective:**

To assess changes in the percentage of applicants to OBGYN residency programs by state based on abortion restrictions in place after the *Dobbs v Jackson Women’s Health Organization* (hereafter, *Dobbs v Jackson*) US Supreme Court decision and examine whether applicants’ preference for programs, as suggested by the distribution of application signals that express higher interest, was associated with abortion bans.

**Design, Setting, and Participants:**

This serial cross-sectional study used anonymized data for all applicants to OBGYN residency programs in the US during September and October from 2019 to 2023. Data were obtained from the Association of American Medical Colleges Electronic Residency Application Service.

**Exposures:**

Applications and program preference signals sent to OBGYN residency programs, analyzed by applicants’ self-reported demographics.

**Main Outcomes and Measures:**

The primary outcome was differences in the percentage of unique applicants to OBGYN residency programs from 2019 to 2023, with programs categorized by state-based abortion restrictions after the *Dobbs v Jackson* decision. Secondary outcomes included the distribution of program signals by state abortion ban status.

**Results:**

A total of 2463 applicants (2104 [85.4%] women) who applied to OBGYN programs for the 2023 residency match cycle were the focal sample of this study. While overall applicant numbers remained stable between 2019 and 2023, the number of applicants differed significantly by state abortion ban status in the 2022 (*F*_2,1087_ = 10.82; *P* < .001) and the 2023 (*F*_2,1087_ = 14.31; *P* < .001) match cycles. There were no differences in the number of signals received by programs in states with bans after controlling for known covariates such as number of applications received and program size, and there were no differences in the percentage of signals sent by out-of-state applicants to programs in states with different abortion laws than their home states (*F*_2,268_ = 2.41; *P* = .09).

**Conclusions and Relevance:**

In this cross-sectional study, there was a small but statistically significant decrease in the number of applicants to OBGYN residency programs in states with abortion bans in 2023 compared with 2022. However, applicant signaling data did not vary by states’ abortion ban status. While OBGYN residency programs almost completely filled in 2023, continued monitoring for the potential consequences of state abortion bans for OBGYN training is needed.

## Key Points

**Question:**

Has there been a change in the percentage of applicants to obstetrics and gynecology (OBGYN) residency programs in states with strict abortion laws?

**Findings:**

In this cross-sectional study of 2463 OBGYN residency program applicants, between 2022 and 2023, there was a small but significant decrease in the percentage of applicants to programs in states with stricter abortion laws.

**Meaning:**

The findings suggest early evidence of a decline in the number of unique applicants to OBGYN residency programs in states with strict abortion laws.

## Teaser

his cross-sectional study assesses changes in the percentage of applicants to gynecology residency programs in states that implemented abortion restrictions after the US Supreme Court's *Dobbs v Jackson Women’s Health Organization* decision.

## Introduction

On June 24, 2022, in the *Dobbs v Jackson Women’s Health Organization* (hereafter, *Dobbs v Jackson*) decision, the US Supreme Court overturned *Roe v Wade*, which held that women had a fundamental right to an abortion.^1^ As a result of this decision, each state has the authority to implement abortion policies without concern for constitutional challenge. Abortion banning and restriction affects the training of 43.9% of residents in US residency programs in obstetrics and gynecology (OBGYN).^2^ A concern raised by educational leaders, residency programs, and applicants to OBGYN is whether access to training in comprehensive women’s health care would alter applications to programs in states with abortion restrictions and bans. Additionally, future workforce concerns are part of a growing national conversation,^3^ as roughly half of residency graduates typically practice in the state where they train.^4^

Simultaneously, matching into an OBGYN residency program in the past decade has become more competitive. Between 2013 and 2023, the number of applicants applying to categorical OBGYN positions increased by 62%, from 1498 to 2433,^5^ while the number of postgraduate year 1 positions only increased by 19%, from 1259 to 1503.^6^ Applicants have responded to this increased competition by applying to more programs; the mean number of applications submitted by each applicant increased from 33 in 2013 to 75 in 2023.^7^ This increase may have made it challenging for program directors to gauge applicants’ true interest in their program and to holistically review all the applications they receive.

One intervention to address the increase in the number of applications submitted and to allow applicants to express a higher level of interest to program directors in a particular program was the implementation of program signals during the 2022-2023 residency match cycle. Applicants were permitted to send up to 18 signals—3 gold signals to indicate their most preferred programs and 15 silver signals to indicate more preferred programs—as part of a supplemental application submission through the Association of American Medical Colleges (AAMC) Electronic Residency Application Service (ERAS).

The primary objective of this study was to assess changes in the percentage of applicants to OBGYN residency programs, with programs categorized by state-based abortion restrictions in place after the *Dobbs v Jackson* decision. Additionally, this work sought to assess whether applicants’ preference for programs, as suggested by the distribution of program signals, was associated with abortion bans.

## Methods

In this serial cross-sectional study, anonymized applicant and program data were obtained from ERAS. Institutional review board approval and informed consent were not required due to the use of deidentified, non–Health Insurance Portability and Accountability Act data. The study was classified as exempt and was not regulated by the University of Michigan Medical School institutional review board. This study followed the Strengthening Reporting of Observational Studies in Epidemiology (STROBE) reporting guideline.

### Data and Study Population

The data were provided by applicants to ERAS during the months of September and October from 2019 to 2023 (applications outside this time frame were excluded) and included self-reported degree (doctor of medicine [MD], doctor of osteopathic medicine [DO], or international medical graduate [IMG]); self-reported gender; permanent address (home state), which was used as a proxy for the home state analyses given that there is not any other means of ascertaining home state in the ERAS package; and self-reported race and ethnicity using categories listed on the ERAS application (African American or Black, Asian, Native American or Pacific Islander, White, other, or prefer not to say). Race and ethnicity were included in the analysis to check for any behavioral differences among groups. A generated variable of underrepresented in medicine (URiM) was defined per the AAMC^8^ (self-identification as 1 or more of the following racial and ethnic categories: American Indian or Alaska Native; Black or African American; Hispanic, Latino, or of Spanish origin; or Native Hawaiian or Other Pacific Islander). Non-URiM was defined as anyone who self-identified as only White or Asian. Those who self-identified as other race and ethnicity alone were not included in these analyses.

Program size was included as a covariate in the analysis. Programs self-reported the number of graduate medical education (GME) positions available in the 2022 match cycle using the 2022 National GME Census in GME Track.^9^

States were grouped into 1 of 3 categories (abortion ban, gestational limit, or no ban) based on laws concerning abortion in effect as of September 9, 2022.^10^ We based our analysis on the *New York Times* designations,^10^ given that applicants needed to make decisions based on the information that was readily available for them in September 2022. This date was chosen because program signaling closed on September 16, 2022, and over 95% of MD and DO graduates and over 90% of IMG graduates applied in September across included application cycles. A generated variable for home state ban status indicated the ban status of the state of an applicant’s permanent address on September 9, 2022. All IMG applicants were excluded from this variable.

### Statistical Analysis

For our primary outcome, we examined the percentage of unique applicants who applied to 1 or more programs in states categorized by ban status for the match cycles from 2019 to 2023. If applicants applied to multiple programs with the same ban status in the same year, their applications were counted once; if applicants applied to multiple programs in states with different ban statuses, their applications were counted once in each ban status. Descriptive statistics, including frequencies, percentages, means, SDs, and repeated-measures analysis of variance (ANOVA)^11^ were used to explore the differences in application volume, followed by post hoc tests with Bonferroni adjustment to further explore the source of differences. Because this was an exploratory study, pairwise comparisons were conducted in the post hoc tests. We conducted a repeated-measures ANOVA at the program level with year as the repeated factor and ban statuses as the grouping variables to assess whether changes in the number of applicants across years and abortion ban statuses were significantly different. In the program-level analysis, the number of applications was counted for each program, and if an applicant submitted multiple applications to different programs in the same year, all their applications would be included in the sample for each program to which they applied. Secondary analyses examined the percentage of in-state (home state) and out-of-state applicants who applied to 1 or more programs in states categorized by ban status.

We also examined the distribution of program signals to states categorized by ban status. Descriptive statistics, including frequencies, percentages, means, SDs, and analysis of covariance (ANCOVA)^11^ conducted at the program level were used to explore the differences in program signals. Program size was used as a covariate in the ANCOVA because programs that received more applications also received more signals. Further analyses examining differences in the percentage of applicants who sent program signals subset by gender and URiM status were performed. Additional investigation was performed on program signals received from in-state vs out-of-state applicants using 1-way ANOVA^11^ to understand whether the mean percentage of program signals from out-of-state applicants differed in ban status groups. The percentage of out-of-state applicants was calculated for each program in this step. All analyses were conducted using R, version 4.2.3 (R Project for Statistical Computing), and statistical significance was set at *P* < .05 (2-tailed). Analyses were conducted separately for gold and silver signals and for combined total signals. Results did not differ for gold and silver signals; therefore, results are presented for the combined analysis only.

## Results

The 2463 unique applicants who applied to 292 OBGYN programs for the 2023 cycle were the focal sample of this study, which included data from the 2019 to 2023 cycles (346 [14.0%] men; 2104 [85.4%] women). A total of 1624 applicants (65.9%) were non-URiM (495 [20.1%] Asian, 1129 [45.8%] White), and 695 (28.2%) were URiM (22 [0.9%] American Indian or Alaska Native; 357 [14.5%] Black or African American; 336 [13.6%] Hispanic, Latino, or of Spanish origin; and 5 [0.2%] Native Hawaiian or Other Pacific Islander). A total of 24 694 applications from 3328 unique applicants were excluded because they were submitted outside the specified time frame.

In September 2022, at the time of application submission for the 2023 match cycle, 11 states had fully enacted abortion bans and 6 others had gestational limits.^1^ As shown in Table 1, while the overall number of OBGYN applicants between 2019 and 2023 remained stable, residency programs in states with bans and gestational limits received fewer unique applicants than did those in states without bans, and the overall mean number of applications received by programs in states with different ban statuses varied in different years (*F*_8,1087_ = 17.99; *P* < .001). Post hoc analysis results showed that the number of applicants to programs differed significantly by abortion ban status in the 2022 (*F*_2,1087_ = 10.82; *P* < .001) and 2023 (*F*_2,1087_ = 14.31; *P* < .001) match cycles. This difference was greater for MD applicants. By studying the descriptive statistics, the percentage of MD applicants who applied to at least 1 program in states with abortion bans decreased to 81.9% (1170 of 1429) in the 2023 match cycle compared with prior years, in which the percentage was stable, ranging from 85.9% (1300 of 1513) in 2022 to 88.0% (1260 of 1432) in 2021. The percentage of unique applicants who applied to programs in states with a different abortion ban status than their home state remained stable in gender groups across years.

**Table 1.** Unique Applicants Who Submitted 1 or More Applications to Programs in States With Abortion Bans, Gestational Limits, and No Bans for the 2019-2023 Match Cycles^a^

Cycle	Applicants, No./total No. (%)		
	States with ban	States with gestational limit	States with no ban
**All**			
2019	2120/2539 (83.5)	2281/2539 (89.8)	2511/2539 (98.9)
2020^b^	2041/2460 (83.0)	2203/2460 (89.6)	2446/2460 (99.4)
2021^b^	2109/2501 (84.3)	2296/2501 (91.8)	2471/2501 (98.8)
2022^b^^,^^c^	2185/2580 (84.7)	2382/2580 (92.3)	2558/2580 (99.1)
2023^b^^,^^c^	2029/2463 (82.4)	2245/2463 (91.1)	2447/2463 (99.4)
**MD**			
2019	1246/1427 (87.3)	1338/1427 (93.8)	1417/1427 (99.3)
2020	1235/1427 (86.5)	1312/1427 (91.9)	1424/1427 (99.8)
2021	1260/1432 (88.0)	1350/1432 (94.3)	1421/1432 (99.2)
2022^b^^,^^d^	1300/1513 (85.9)	1420/1513 (93.9)	1504/1513 (99.4)
2023^b^^,^^d^	1170/1429 (81.9)	1326/1429 (92.8)	1425/1429 (99.7)
**DO**			
2019	426/478 (89.1)	455/478 (95.2)	467/478 (97.7)
2020	365/411 (88.8)	388/411 (94.4)	408/411 (99.3)
2021	377/426 (88.5)	406/426 (95.3)	422/426 (99.1)
2022	459/504 (91.1)	481/504 (95.4)	501/504 (99.4)
2023	417/460 (90.7)	437/460 (95.0)	458/460 (99.6)
**IMG**			
2019	448/634 (70.7)	488/634 (77)	627/634 (98.9)
2020^b^	441/622 (70.9)	503/622 (80.9)	614/622 (98.7)
2021^b^	472/643 (73.4)	540/643 (84)	628/643 (97.7)
2022	426/563 (75.7)	481/563 (85.4)	553/563 (98.2)
2023^b^	442/574 (77.0)	482/574 (84.0)	564/574 (98.3)

Abbreviations: DO, doctor of osteopathic medicine; IMG, international medical graduate; MD, doctor of medicine.

^a^ Ban status after the *Dobbs v Jackson Women’s Health Organization* decision is shown.

^b^ Significant difference between states with a total abortion ban vs no ban, after Bonferroni correction.

^c^ Significant difference between states with gestational limits vs no abortion ban, after Bonferroni correction.

^d^ Significant difference between states with gestational limits vs total abortion ban, after Bonferroni correction.

Further investigation of MD and DO applicants showed that the decrease in the percentage of unique MD applicants applying to programs in states with abortion bans in the 2023 cycle was associated with the abortion ban status in applicants’ home states. As shown in Table 2, a smaller percentage of unique MD applicants from states without bans applied to 1 or more programs in states with complete bans than in the prior cycle (733 of 925 [79.2%] in 2022 vs 641 of 875 [73.3%] in 2023).

**Table 2.** Unique US MD and DO Applicants Who Submitted 1 or More Applications to Programs in States With Abortion Bans, Gestational Limits, and No Bans by Abortion Law in the Applicant’s Home State, 2019-2023 Match Cycles^a^

Applicant home state ban status	Applicants, No./total No. (%)		
	States with ban	States with gestational limit	States with no ban
**MD**			
2019			
Ban	306/309 (99.0)	295/309 (95.5)	304/309 (98.4)
Gestational limit	244/257 (94.9)	256/257 (99.6)	253/257 (98.4)
No ban	687/850 (80.8)	777/850 (91.4)	849/850 (99.9)
2020			
Ban	280/281 (99.6)	277/281 (98.6)	279/281 (99.3)
Gestational limit	228/237 (96.2)	235/237 (99.2)	236/237 (99.6)
No ban	718/899 (79.9)	791/899 (88.0)	899/899 (100)
2021			
Ban	281/281 (100)	273/281 (97.2)	274/281 (97.5)
Gestational limit	251/268 (93.7)	268/268 (100)	265/268 (98.9)
No ban	718/873 (82.2)	799/873 (91.5)	872/873 (99.9)
2022			
Ban	288/290 (99.3)	287/290 (99.0)	286/290 (98.6)
Gestational limit	272/289 (94.1)	286/289 (99.0)	285/289 (98.6)
No ban	733/925 (79.2)	838/925 (90.6)	924/925 (99.9)
2023			
Ban	267/269 (99.3)	265/269 (98.5)	268/269 (99.6)
Gestational limit	254/277 (91.7)	276/277 (99.6)	274/277 (98.9)
No ban	641/875 (73.3)	777/875 (88.8)	875/875 (100)
**DO**			
2019			
Ban	95/95 (100)	87/95 (91.6)	89/95 (93.7)
Gestational limit	98/105 (93.3)	105/105 (100)	101/105 (96.2)
No ban	229/274 (83.6)	259/274 (94.5)	273/274 (99.6)
2020			
Ban	96/97 (99.0)	95/97 (97.9)	95/97 (97.9)
Gestational limit	68/74 (91.9)	74/74 (100)	73/74 (98.6)
No ban	198/237 (83.5)	216/237 (91.1)	237/237 (100)
2021			
Ban	106/108 (98.1)	103/108 (95.4)	105/108 (97.2)
Gestational limit	79/86 (91.9)	85/86 (98.8)	85/86 (98.8)
No ban	188/227 (82.8)	213/227 (93.8)	227/227 (100)
2022			
Ban	111/112 (99.1)	110/112 (98.2)	111/112 (99.1)
Gestational limit	92/100 (92.0)	98/100 (98.0)	99/100 (99.0)
No ban	252/288 (87.5)	269/288 (93.4)	287/288 (99.7)
2023			
Ban	106/107 (99.1)	101/107/ (94.4)	105/107 (98.1)
Gestational limit	97/102 (95.1)	101/102 (99.0)	102/102 (100)
No ban	211/248 (85.1)	232/248 (93.5)	248/248 (100)

Abbreviations: DO, doctor of osteopathic medicine; MD, doctor of medicine.

^a^ Ban status after the *Dobbs v Jackson Women’s Health Organization* decision is shown.

**Figure 1.**
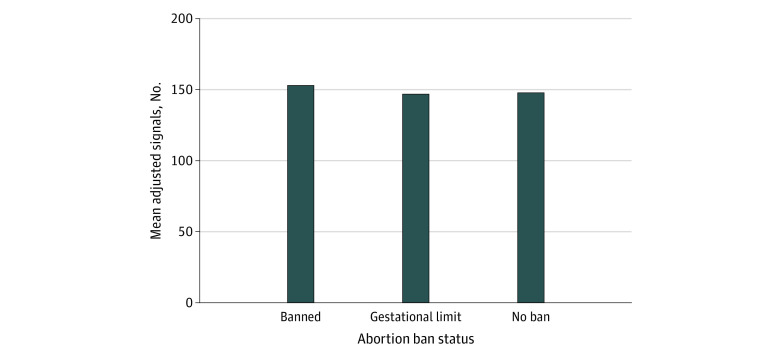
Mean Adjusted Number of Program Signals Received by Obstetrics and Gynecology Programs in States With Abortion Bans, Gestational Limits, and No Bans in the 2023 Match Cycle The mean adjusted number of program signals within each ban status category was computed after controlling for covariates (number of applications received and program size) through the least-squares means in the analysis of covariance model.

**Figure 2.**
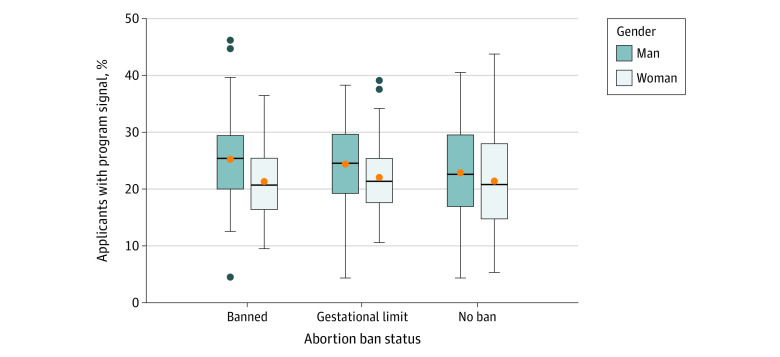
Percentage of Program Signals Sent to Obstetrics and Gynecology Programs in States With Abortion Bans, Gestational Limits, or No Bans in the 2023 Match Cycle, by Applicants’ Gender The horizontal bar inside the boxes indicates the median percentage of applicants with program signals across programs (N = 283) and the lower and upper bounds of the boxes, the first and third quartiles. The whiskers indicate the largest values no further than 1.5 times the IQR from the ends of the boxes, and data more extreme than the whiskers are plotted individually as outliers (black dots). Orange dots indicate the mean percentage of applicants with program signals across programs.

**Figure 3.**
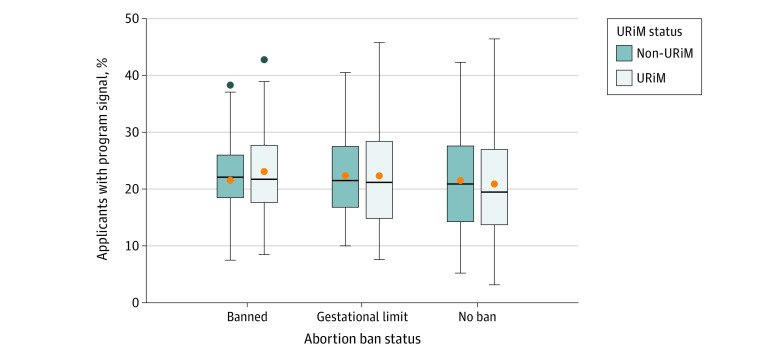
Percentage of Program Signals Sent to Obstetrics and Gynecology Programs in States With Abortion Bans, Gestational Limits, or No Bans in the 2023 Match Cycle, by Applicants’ Underrepresented in Medicine (URiM) Status The horizontal bar inside the boxes indicates the median percentage of applicants with program signals across programs (N = 283) and the lower and upper bounds of the boxes, the first and third quartiles. The whiskers indicate the largest values no further than 1.5 times the IQR from the ends of the boxes, and data more extreme than the whiskers are plotted individually as outliers (black dots). Orange dots indicate the mean percentage of applicants with program signals across programs.

During the 2023 match cycle, 2352 of the 2463 OBGYN applicants (95.5%) provided program signals. While there were significant differences in the number of signals received by programs (*F*_4,258_ = 214.47; *P* < .001), the differences could be explained by known covariates of program signals, the number of applications received, and the program size rather than ban status (Figure 1). Programs with more applicants and with more available postgraduate year 1 positions received more signals. Ban status was not associated with received signals after controlling for those known covariates. In addition, as shown in Figure 2 and Figure 3, there was no noticeable difference in the percentage of program signals received in each ban category when stratifying applicants by gender and URiM status. Furthermore, programs in states with abortion bans did not receive a significantly smaller percentage of program signals from out-of-state applicants compared with states without bans. One-way ANOVA including all applicants at the program level showed no statistically significant difference in the percentage of program signals sent by out-of-state applicants to programs with different abortion laws than their home state (*F*_2, 268_ = 2.41; *P* = .09).

## Discussion

To our knowledge, this is the first examination of applicants to OBGYN residency in the match cycle immediately after landmark changes in abortion legislation in 2022, in the midst of the simultaneous implementation of the program signaling initiative. A small decrease in the percentage of applicants who applied to residency programs in states with abortion bans and restrictions was observed. This difference was significant between the 2022 and 2023 match cycles. When controlled for covariates of program signaling, including numbers of applications received by programs and program size, numbers of program signals received did not vary significantly based on state ban status. These observations must be viewed in the context of continued successful placement of applicants into residency programs, as evidenced by low numbers of unfilled programs in the specialty from 2019 to 2023, which ranged from 3 to 4 categorical open positions.^12^

As has been previously described,^13^ the potential workforce and health equity repercussions will be immense if fewer physicians decide to practice in states with abortion restrictions and bans, especially given that there are already fewer physicians practicing in many of these areas.^14^ However, we must exercise caution and carefully interpret available data in the context of changes in application process initiatives. It is unclear just what, if any, impact a small decrease in applicants to programs in states with abortion restrictions will have, and this warrants close follow-up. Given that the goal of the program signaling initiative was to enable applicants to demonstrate their genuine interest amid rising application inflation, our findings in this first year may reflect an indication of true applicant intent and interest in specific programs rather than being reflective of the abortion restrictions. The mean number of applications per applicant in the 2022-2023 match cycle was 75^7^; thus, a small decrease in applicants in states with abortion restrictions may have minimal practical implications. Prior to the 2023 match cycle and the use of signaling, applicants may have been sending applications to programs at which they were unlikely to interview or, if interviewed, were unlikely to rank highly on their match list. It is also important to note that the overall percentage of applicants who applied to programs in states with abortion restrictions and bans was still high (86.8%). Furthermore, as most OBGYN residency positions are consistently filled, the fact that the ultimate match results were not different for states with and without abortion restrictions and bans is an important marker and needs longer-term analyses. The differences noted in this cycle between the behavior of MD applicants and DO and IMG applicants will also need to be followed up in subsequent years to determine whether there are any changes in ultimate distributions of matched residents in states based on abortion restrictions and type of medical school background.

Although it is challenging to make inferences about program signaling patterns in the first year of implementation, the mean number of signals sent to programs in states with abortion restrictions and bans was similar to that in states without restrictions and bans. This may imply that applicants were aware that all training programs are required by the Accreditation Council for Graduate Medical Education to provide in-person training in abortion care if a resident desires training, which might have affected their choices. Regardless, program signaling trends will be important to follow and examine in subsequent cycles for a more accurate indication of applicant preference and intent.

Obstetrics and gynecology remains a competitive specialty, with most residency positions filled with highly qualified applicants. The ongoing challenges to comprehensive training in full-breadth, evidenced-based OBGYN care imposed by state abortion bans and gestational limits bear close and careful monitoring for any continued and potential other consequences for applicant behaviors and patterns. Further research into these consequences may prove to be critically important to both the future workforce of OBGYN physicians in the US and the patients and diverse populations they serve.

### Limitations

A limitation of this study was the challenge in interpreting changes in applicant data with the simultaneous implementation of the new program-signaling initiative amid the changes in abortion access by state. Program signaling was new, and programs and applicants were still in the process of familiarizing themselves with how best to use program signals. In addition, the changing landscape of abortion restrictions, along with the potential confusion that these changes might have caused among applicants and how soon they were aware of new information and legislation, could have affected their choices in the 2023 cycle. Furthermore, our analyses had statistical limitations. The data in this study were aggregated at the program level to conduct statistical analyses using repeated-measures ANOVA, ANCOVA, and 1-way ANOVA. While we controlled for program size with the ANCOVA analysis, no other program-level variables were controlled for in the other analyses. In addition, even though there were few missing data at the program level and ANOVA models could work properly from this perspective, the same applicants could send applications or program signals to different programs, which could be better handled by mixed models. Future research could use mixed models to better control for clustering. Lastly, as this was an exploratory study, we performed omnibus tests for an overall difference across the groups followed by post hoc pairwise comparisons to assess sources of difference. Future studies could focus on planned comparison tests when clearer hypotheses are available to increase the statistical power of each comparison.

## Conclusions

This cross-sectional study found a small but statistically significant decrease in the number of applicants to OBGYN residency programs in states with abortion bans and restrictions implemented after the *Dobbs v Jackson* decision. However, the number of application signals received by programs did not vary by state ban status, and residency programs continued to fill. While OBGYN residency programs filled the vast majority of available positions in 2023 as in previous years, continued monitoring for potential consequences of state bans in terms of applicant behaviors will be critical.

## Notes

Supplement.
Data Sharing Statement
