# Supplementary material for: Trends in Obstetrics and Gynecology Residency Applications in the Year After Abortion Access Changes
Source: JAMA Netw Open. 2024 Feb 7;7(2):e2355017. doi: 10.1001/jamanetworkopen.2023.55017 (PMC10851098; doi:10.1001/jamanetworkopen.2023.55017)
Supplement: Supplement. — Data Sharing Statement [file jamanetwopen-e2355017-s001.pdf]

Data Sharing Statement

Hammoud. Trends in Obstetrics and Gynecology Residency Applications in the Year After Abortion Access Changes. *JAMA Netw Open*. Published February 07, 2024. doi:10.1001/jamanetworkopen.2023.55017

Data
Data available: No
